# Long experience with a web-based, interactive, conversational virtual patient case simulation for medical students’ evaluation: comparison with oral examination

**DOI:** 10.1080/10872981.2021.1946896

**Published:** 2021-06-28

**Authors:** Arie Oliven, Rachel Nave, Adam Baruch

**Affiliations:** Technion - Israel Institute of Technology, Haifa, Israel

**Keywords:** Virtual patient, medical education, e-learning, assessment, clinical competence

## Abstract

Virtual patients (VP) have been advocated as reliable tools for teaching and evaluating clinical skills and competence. We have developed an internet-based, OSCE-like, conversational VP system designed both for training and assessment of medical students. The system, that encompasses complete patient management from H&P to diagnostic procedures and treatment, has now been used regularly during the clerkship of internal medicine. The present article describes the system and compares assessments undertaken with the VP-system over the last five years, to traditional bed-side oral exams. All students practiced on their own exercise VP cases, while preparing for the final exam. A total of 586 students were evaluated simultaneously with both assessment modalities. The αCronbach of the VP exam averaged 0.86. No correlation was found between the grades obtained in the two exams, indicating that the VP exam evaluated different parameters than those assessed by the examiners in the oral examinations. We conclude that a VP system can be utilized as a valid and reliable examination tool. It is also most useful for independent training by students during their ward-based learning, as well as when not studying in classes, wards or clinics, when social distancing is required.

## Introduction

Modern health care system is compelled to translate advances in technology, basic and clinical sciences into medical practice. In parallel, modern medical education must translate these advances into educational practice. The rapidly expanding medical knowledge demands more efficient teaching methods and the teaching goals of medicine emphasize the need to develop clinical thinking and patient management skills more than impart knowledge. However, lectures and book learning remain the primary means of learning for most students, as most exams require predominantly knowledge of facts.

Bedside teaching is undoubtedly the most important element of clinical education. Unfortunately, it requires a large core of experienced and well trained instructors, and the number of adequate patients available for teaching, as well as the spectrum of diseases that students can be exposed to during a rotation are insufficient. Similarly, bed-side oral examination based on real patients are known to have unacceptable low reliability, and were often replaced by OSCE (objective structured clinical examination) simulations by actors or medical staff. However, OSCEs are resource intensive [1], and cannot be used routinely by many faculties of medicine. Also, when OSCE evaluators are not well trained and/or are not used on a frequent and continuous basis, their reliability and validity may be jeopardized [2,3].

The incorporation of computing technology into medical education offers the promise of addressing educational challenges in new ways [4–6]. The advance of computerized systems has enabled to develop a new level of tuition and evaluation, often defined as virtual patient (VP), that can be placed between the book and written exams on the one hand, and bedside teaching and assessment on the other. The term VP has been used in various contexts [7,8]. An often-cited definition was proposed by the American Association of Medical Colleges, that delineated VP as a software that simulates real-life clinical scenarios, enabling to obtain history and physical (H&P) and make diagnostic and therapeutic decisions [9]. The advantages of a web-based VP system for tuition and practice is obvious, as students can use the software for interactive learning also at home, and no installation, setup or maintenance are required. Its advantages for testing are clear: full objectivity (the same ‘examiner’ and the same questions for all students without bias by appearance and speaking skills) and high reliability when using an appropriate number of clinical cases and questions.

In our faculty we developed and implemented 12 years ago a VP software designed in the first stage to resemble the H&P part of OSCE, and entitled VP level-I. Unique to this software is being conversational, providing the students the possibility to conduct a free dialog with the VP, based on natural language processing, with a lexicon of keywords. The student writes medically relevant questions in free wording and the VP analyses the questions. It leverages remarkable understanding, providing the adequate predetermined reply to many formulations of a question. This feature is novel and fills an important gap in the design of other VP computer programs: to the best of our knowledge, even to date all VP programs that are in use base the history part on selecting questions from a limited question-database. This approach provides of course inappropriate hints, and is not at all an alternative to questioning a simulated patient. Similarly, when using our VP software, unlike others, students are required to ask/write in their own words if specific signs of physical examination relevant for the case are present. This approach indicates if the student knows, independently and without hints, which signs characterize the patient’s disease as well as those of its differential diagnosis, and should be looked for. The answer given by the VP software may be a written reply (like for palpation findings), a picture (for visual findings), or audio-video clips of heart and lung sounds, that the student has to interpret. The VP software was well accepted by the students [10]. The VP level-I software was implemented in the H&P course, and used initially for 3 years in parallel with conventional OSCE with actors. The comparison of these assessment modalities was found to provide similar grades, with better reliability (higher α Cronbach values) of the VP test [11]. Following these findings we stopped using OSCE, which is much more expensive and cumbersome compared to the computerized VP.

Subsequently, the level-I VP software was upgraded to resemble a realistic, more comprehensive patient encounter, covering, in addition to H&P, also laboratory and imaging and other diagnostic procedures, and therapy (i.e., complete ‘patient management’), from presentation to treatment. For this purpose, lists of laboratory tests, imaging modalities and other diagnostic tests were introduced, similar to computerized order lists available in hospitals and other health agencies. Our primary goal was to teach more advanced student how to evaluate and manage patients with specific common clinical conditions, and improve their clinical skills. The required items, presented as a list of asked/ordered and missing/not-asked items (or incorrectly interpreted, like erroneous auscultatory or x-ray findings) for each case and patient management part (history, laboratory etc.), provide the feedback of our training VP modality. In addition, at the end of each case practice, the student gets a grade that rates his current achievement and knowledge in the particular field. New cases were created each year, and earlier cases used in previous years’ exams were open to the students for exercise and self-assessment.

The VP level-II examination could be criticized as being redundant, since medical teachers examine its main topics (patient management, reasoning, integration of findings and the like) for many years. We hypothesized that despite the similarity in the test objectives, the two forms of examination are influenced by different factors and accordingly their results will not be similar. Therefore, the aim of the present study was to compare the two modes of evaluation, using our unique local setting where all students are tested on the same clinical material, one day apart in each of the test modalities, at the end of the internal medicine clerkship.

## Methods

Medical studies in our faculty last six years. The fourth year is the first clinical year and is devoted almost entirely to internal medicine.

The H&P course is one of the courses of the first semester, and at the end of this course a VP level-I test is held. The second semester is mostly devoted to internal clerkship. At the end of this semester, on consecutive days, a traditional bedside oral examination and the VP level-II examination (same software as above but with the practice feedback blocked) are performed, as part of the students’ assessment modalities. The curriculum remained unchanged throughout the period relevant to this work. For the purpose of comparing the VP level-II to the oral examination we have used the final scores of the two exams over the last 5 years, from the inclusion of the VP level-II exam in our faculty curriculum until the routine course of learning and evaluation was disrupted by the Covid-19 pandemic. IRB approval was waived as all data used for this study was accessible to the authors by virtue of their role in the faculty, and only they performed the data analysis.

The VP exams were conducted in a computer classroom of the Faculty of Medicine of the Technion, Haifa, Israel. Much emphasis was placed on exam security: testing runs with a ‘lock-down’ browser, and strict identity verification of the examinees was undertaken. In each exam, 5 virtual cases were presented sequentially, the same (but in variable order) to all students. The number of items required in each case varied, and accordingly the time assigned for each VP case was different from case to case, ranging between 25–45 min. Most items were scored as 1 point, but some items (like interpretation of auscultatory findings and ECG or some cardinal treatments) were scored higher (2–3 points). In each case, the students were required to go one by one on 5 steps: history, physical examination, laboratory tests, imaging and other diagnostic procedures, and treatment. When the pre-determined time elapsed, the computer automatically switched to the next case. The test results, including analysis of the difficulty of each item and its ability to differentiate between better and weaker students (point biserial correlation coefficient, rpb), and the reliability of the whole exam (Cronbach’s alpha), are produced automatically at the end of the exam.

Oral bed-side exams were conducted in the departments of internal medicine, by clerkship tutors that evaluated students they did not teach. The tutors/examiners were mostly attending physicians of the department of internal medicine, and in part 4th (last) year residents. Adequate patients were selected in advance. Each student was assessed by one examiner, based on the management of one patient, and each examiner evaluated one by one 3–4 students. After providing some information about the patient, the examiner observed how the student was taking history and performing physical examination. Thereafter, the student was given results of laboratory tests and imaging that he requested. In this context, the student could be asked to describe a chest x-ray or ECG. The student was also asked to provide differential diagnosis, and at the end to suggest the adequate treatment. As the entire exam was limited to 30–45 minutes for each student, only a portion of the above was actually done. H&P were truncated, only part of the laboratory and imaging results were discussed etc. The examiners were asked to assess each part of patient evaluation they examined and to provide a final grade based on these assessments.

Data analysis: In the VP exam, each correct H&P question, test or treatment earned the student 1–3 points, and his total score was his total number of points divided by the number of points that could be obtained in the exam, presented as percentage. The software provided multiple individual and group average data and results. For the purpose of this study we used the individual total scores of the whole exam and those of the single parts of patient evaluation (history, physical etc.). These data were used to calculate the average scores of the class each year. In the oral exams, only final grades were available for all students. Comparison of the scores in the different parts of the VP exam (history, physical examination etc.), and the comparison of the scores in the VP and oral exams were carried out using t-test. Pearson correlation was used to correlate grades of all students obtained in the VP and oral exams. Data are presented as mean±SD. p < 0.05 was considered as statistically significant.

## Results

Over the five years, 586 students were examined. The scores that could be reached for each VP case ranged from 25 to 68, with a total score of 206–277 points obtainable in each exam. These scores were converted to grades between 0–100, and a grade of 70 was required to pass the exam. Cronbach’s alpha of the exams averaged 0.86, with 0.89 obtained both in the first (2015) and the last (2019) exam.

Table 1 presents the grades obtained for each section of the VP cases in the consecutive years. The scores for history and treatment were consistently lower than those obtained for lab tests and other diagnostic procedures, although differences tended to decrease in recent years. In addition, the final grades in the VP exams in the first two years were lower than in the following 3 years (p < 0.05).

**Table 1.** Comparison of the average grades obtained by the students for each of the 5 sections of VP case management

year	history	physical	lab	imaging*	treatment
2015	74.6	80.0	80.8	85.8	73.2
2016	71.2	68.0	85.0	84.0	72.6
2017	81.4	86.4	91.6	89.2	85.6
2018	82.2	83.0	87.4	86.2	83.8
2019	84.8	85.8	86.8	86.4	82.8
mean	78.8 ± 5.7^1^	80.6 ± 7.5	86.3 ± 3.9	86.3 ± 1.9	79.6 ± 6.2^1^

* – including additional diagnostic procedures.

1 – p < 0.01 for comparison with laboratory and imaging items.

Table 2 compares average grades obtained each year in the VP and oral examinations. It can be seen that the grades given by the tutors to the students every year in the oral exams were significantly higher than those obtained by the same students in the computerized VP exam.

**Table 2.** Average grades of the students in the VP and oral examinations

year	VP exam	Oral exam
2015	82.2^1^	91.9^4^
2016	82.6^2,3^	91.1^4^
2017	86.0	91.2^4^
2018	84.9	91.7^4^
2019	84.3	91.2^4^
mean±SD	84.3 ± 1.6	91.2 ± 0.4

1 – p < 0.01 for comparison with 2017, 2018 and 2019.

2 – p < 0.01 for comparison with 2017.

3 – p < 0.05 for comparison with 2018 and 2019.

4 – p < 0.001 for comparison with VP exam.

**Figure 1.**
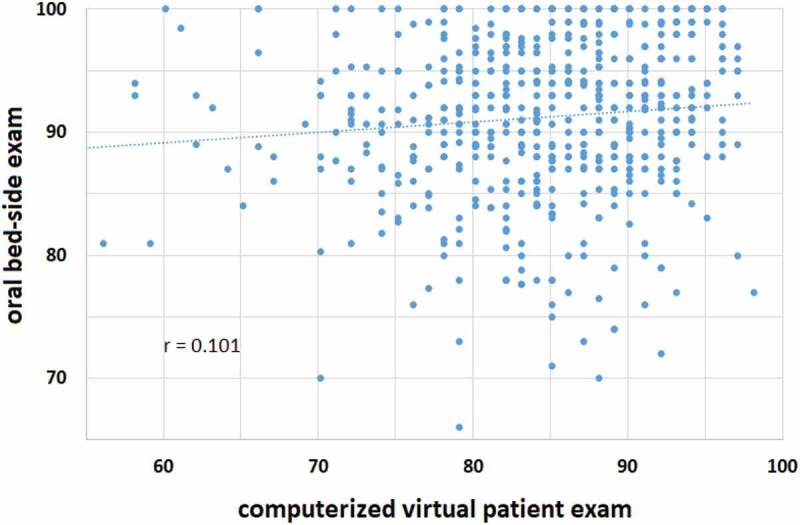
Relationship between the grades obtained by all students (n = 586) in the VP exam (x-axis) and the oral exam (y-axis)

The relationships between individual grades obtained by all students in the two exams over the 5 years is shown in Figure 1. Pearson’s correlation coefficient (r) of this relationship was 0.101, indicating that, although statistically significant (p < 0.05), there was only a minimal relationship between the grades obtained in these two modalities of assessment. As seen in the figure, 17 students (3%) failed the VP exam (2–5 each year), as compared to a single student in the oral exam. The correlation between the grades of the two exams in each one of the five years ranged between 0–0.24, reaching statistical significance (p < 0.05) in only two of the five years.

## Discussion

Over recent years, an increasing number of computer-based patient simulations have been proposed for both training and assessment in medical education [12]. Although the use of VPs has not yet entered the main stream of medical curriculum [13], there is no doubt in the potential of VPs to fill significant gaps in the current instruction of medical students. VPs are excellent teaching tools for developing clinical reasoning and decision-making skills and improving clinical competency [14,15]. Clinical reasoning is a process that matures through deliberate practice with multiple and varied clinical cases. VPs are ideally suited to this task, as potential variations in VP design are practically limitless [16]. VPs can incorporate images, sound, videos, lab tests and imaging results, both for early medical students [16], and for later years’ courses [14]. In addition, VP based exams were found to have advantages over other assessment modalities evaluating medical competence [17,18]. However, due to practical and logistic limitations, it appears that despite the obvious theoretical advantages of VPs, few if any university has published its experience with a VP system that was implemented into the curriculum and used regularly for teaching and assessment [13]. Review of the relevant literature seems to indicate that most if not all VP systems designed to teach and assess comprehensive patient management were either feasibility or pilot studies [8,17]. Accordingly, data on how best to implement and use VPs in practice are scarce [13].

Most VP systems provide an interactive ‘game’, enabling practicing patient management whenever and wherever convenient for the student. Our VP system has the unique advantage of being conversational and based on open dialog with the simulated patient, a rare feature in the VP literature [19]. At the sections of H&P, the students write open anamnesis questions and inquire about physical examination findings freely, rather than choosing from lists of question options, that provide unavoidable clues (cueing) to the required items [19]. This unique feature makes our VP exam more similar to a conventional oral exam. Therefore, compared to other VP software, it is the only one that enables a meaningful comparison to the complete patient management examination conducted by a medical teacher. Another factor that enabled a more reliable comparison is the vast experience we have gained in using the software over years, which has made the VP a more ‘experienced examiner’: multiple improvements were introduced also by introducing a machine-learning-like modality, and more than 200 VP cases were compiled.

The VP level-II provides not only an easily accessible and moldable platform to practice and evaluate clinical reasoning, diagnostic skills and decision making [20], but it is also an excellent tool to emphasize patient safety, a topics that is often somewhat neglected by many of the teachers and examiners. For example, prescribing a drug that the patient is allergic to gives a negative score, as does unnecessary invasive tests that may pose the patient at risk. Coagulation tests are required before invasive procedures, renal function tests are mandatory before imaging with contrast material, etc.

As seen in Table 2, although the students were already familiar with the VP level I, few years were required until the average grade of the students stabilized near the middle of the passing range, including the initially relatively low scores for anamnesis and treatment. It is possible that feedbacks passed each year to the teaching departments and discussed in the yearly pre-clerkship workshops of the tutors may have contributed to the improvement, emphasizing the value of objective and reliable feedbacks available when using a VP system. Quite notable was the complete lack of concordance between the scores obtained in the level-II VP and the oral exams. This finding is in line with other works comparing oral bed-side exams with computer-based case simulators [21]. As the oral exams were scored by a single examiner based on the evaluation of a single patient, the results of this exam cannot be extrapolated to oral exams in general. Also, the examiners were asked to lay emphasis on interpersonal communication and the technique of physical examination, topics not evaluated in the VP exam. However, they were instructed to assess the same parameters tested also, one day apart, in the VP exam, i.e., patient management skills based on the knowledge gained at this stage of their studies. Our results suggest that the two exam modalities assess different skills of the students. Of note, throughout the clerkship the students had several multiple choice questions (MCQ) exams, considered to assess primarily clinical knowledge. The correlation of the oral exam scores with the MCQ results was even lower than with the VP exam (r = 0.080). On the other hand, the correlation of the MCQ scores with those obtained in the VP exam was highly significant (r = 0.452, p < 0.0001).

All methods of teaching and assessment have their strengths and intrinsic flaws. Accordingly, several limitations of the VP system need to be addressed: first, our VP does not enable practicing and evaluation of true physical examination and interpersonal communication skills important for doctor-patient relationship. These important skills must be tutored and evaluated by other educational means. Also, while comprising one of the important virtues of our VP system, the use of free communication with the VP (mainly in the section of history questions) is also the most intricate part of our software. The need to enable the VP to understand questions presented in various and often unconventional formulations is the most complex part of creating a new case of a conversational VP. Particularly novice tend to ask irrelevant questions and often use odd formulations [22]. However, once one case has been prepared in a clinical topic, for example a case of jaundice due to hepatitis, the preparation of new cases of jaundice due to other causes is much simpler [23]. As for the students, those who have been practicing many VP cases learn how to formulate simple and clear questions and do not encounter problems during the exam. Also, given the many components of the VP system, it clearly requires some practice before using it for taking an exam. In our opinion, this is one of the advantages of the system: It is generally acknowledged that assessment drives learning [19]; students know that they have to practice a large number of tutorial VP cases in order to be well familiar with the VP system, to avoid difficulties during the exam. Obviously, learning by exercising the practice cases is the main purposes of the VP exam.

In conclusion, the present paper compares the results of oral and VP exams over 5 years, with emphasis on our VP level-II system that applies natural language and is used as a tool to enhance self-learning and assessment of patient management. The VP exam mode integrates all five criteria considered as most valuable for assessment [19]: high reliability and validity, feedback for future learning, easy accessibility and low cost. Comparing bed-side exam grades with those achieved in the VP system suggests that the two assessment tool evaluate different skills. Obviously, books, lectures and conventional bed-side teaching and training remain indispensable. In addition, a comprehensive VP software should complement educational aspects not covered sufficiently by these modalities, providing a most useful, effective and reliable means of teaching, practicing and evaluating skills required for patient management. The availability of the web-based practice modality of the VP in our faculty, that enabled distant learning of patient management, has recently proven itself as a real asset: in view of the required social distancing due to the COVID-19 pandemic and the resulting need to shorten clerkships and to change temporarily the medical curriculum [24], the VP system was added to other e-learning modalities to improve students’ readiness to the clinical rotations.
